# Hierarchy and Psychometric Properties of ADHD Symptoms in Spanish Children: An Application of the Graded Response Model

**DOI:** 10.1371/journal.pone.0164474

**Published:** 2016-10-13

**Authors:** Victor B. Arias, Daniel E. Nuñez, Agustín Martínez-Molina, Fernando P. Ponce, Benito Arias

**Affiliations:** 1 Facultad de Psicología, Universidad de Talca, Talca, Chile; 2 Escuela de Psicología, Pontificia Universidad Católica de Chile, Santiago, Chile; 3 Departamento de Psicología, Facultad de Educación y Trabajo Social, Universidad de Valladolid, España; University Children's Hospital Tuebingen, GERMANY

## Abstract

The Diagnostic and Statistical Manual of Mental Disorders (DSM) diagnostic criteria assume that the 18 symptoms carry the same weight in an Attention Deficit with Hyperactivity Disorder (ADHD) diagnosis and bear the same discriminatory capacity. However, it is reasonable to think that symptoms may differ in terms of severity and even in the reliability with they represent the disorder. To test this hypothesis, the aim of this study was to calibrate in a sample of Spanish children (age 4–7; n = 784) a scale for assessing the symptoms of ADHD proposed by Diagnostic and Statistical Manual of Mental Disorders, IV-TR within the framework of Item Response Theory. Samejima’s Graded Response Model was used as a method for estimating the item difficulty and discrimination parameters. The results showed that ADHD subscales (Attention Deficit and Hyperactivity / Impulsivity) had good psychometric properties and had also a good fit to the model. However, relevant differences between symptoms were observed at the level of severity, informativeness and reliability for the assessment of ADHD. This finding suggests that it would be useful to identify the symptoms that are more important than the others with regard to diagnosing ADHD.

## Introduction

ADHD is a genetic neurodevelopmental alteration characterised by the persistent symptoms of inattention, hyperactivity and impulsivity. These symptoms are maladaptive and incoherent with regard to the child’s level of development. Symptoms can appear before the age of 7. Its clinical manifestations must last longer than 6 months, and the symptoms must be more intense than those observed among other children of the same age, intelligence, and level of development. The symptoms should be present in several contexts and lead to problems in daily life [1]. According to the DSM-IV-TR, the prevalence of ADHD is between 3% and 7%; however, these figures tend to show significant variation depending on the evaluation instruments used, the informants, the study sample size, and other variables [2].

One of the most common ways to evaluate this disorder has been the structured or semi-structured clinical interview [1, 3]. However, evaluations have attempted to be more precise and systematised by transferring the symptomology proposed by the DSM-IV-TR (and recently from DSM-5) to scales and questionnaires. Some of these attempts have been submitted to rigorous psychometric validation (e.g., [4–7]). In general, the validation processes for ADHD evaluations have been conducted following classical test theory (CTT). However, methods based on CTT have important limitations, mainly concerning to the evaluation of each symptom properties and its contribution to the measurement the general construct [8]. For instance, by using CTT is difficult to address the adequacy of certain DSM assumptions, such as the weight of every symptom is similar to make a diagnosis (i.e., all symptoms have identical severity and discriminative capacity). Beyond CTT, methods based on Item Response Theory (IRT, [9]), are specially suitable to evaluate the psychometric properties of an instrument at a symptom level. The advantages of IRT for analysing the psychometric properties of a psychological measurement have been widely discussed [9–17]. Perhaps the most relevant of these advantages includes the following: (a) it is asumed that the actual position of the subject within the latent continuous does not depends on the particular set of used ítems; (b) the effectiveness of the scale can be evaluated at each level of the trait or latent variable; (c) it is possible to estimate the precision with which each test (and each individual item) measures different levels of ability/latent traits of the examined participants; (d) the standard error uses different values throughout the continuum of the latent variable; (e) it allows the calibration of sets of proper items to create adaptive tests; (f) it allows to deeply studying the relationship between each item and the latent variable. In the context of clinical evaluation, the scaling through IRT would allow to determine the relative severity of each symptom (according to the amount of the needed trait for it endorsement). Additionally, the scaling using IRT would allow building highly accurate measurements for high levels of the latent variable (e.g. for screening purposes), and the development of brief questionnaires without losing precision. However, the usage of IRT for clinical assessment implies to solve some issues such as scores with high skewness, the existence of limited sets of indicators, the frequent overlapping among symptoms belonging to different syndromes, and the conceptualization of disorders as quasi-traits [18].

Despite the increasing usage of IRT for assessing non-cognitive symptoms [18], studies using this approach for analyzing ADHD are scant (e.g., [19–25]), as compared to the great amount of studies based on analytic approaches such as confirmatory factor analysis. Overall, IRT-based studies have been focused on the calibration of the scales based on 18 ADHD symptoms in general/community [5, 22–24, 26–28], and mixed clinical/general samples [8, 29], and also on the assessment of differential functioning (DIF) of symptoms considering gender, the age of onset, and the informant (parents and teachers). The main findings of these studies are: (a) as a whole, the psychometric properties of the rating scales based on 18 DSM symptoms are adequate either in the case of parents or teachers; (b) the symptom’s capability to provide accurate information differs along the continuum; (c) the general reliability of the instrument is low for extreme values of the latent variable, and high approximately from the mean to two standard deviations; (d) the symptoms are not equivalent in terms of severity; and (e) certain symptoms seems to function differentially for both gender and age; however because these results vary across studies there is no robust evidence showing that none of them present systematic invariance difficulties.

As mentioned above, few studies have evaluated ADHD symptoms using IRT despite its advantages. Having precise measures is of utmost importance for numerous reasons, among which we highlight the following. First, results from studies testing structural models are as reliable and valid as those from models explaining how latent variables are measured and, by extension, as reliable and valid as the evaluation instruments used. Second, from the perspective of the clinical application of these evaluations, IRT models provide a considerably deeper knowledge of symptoms that ideally provide precise and valid instruments for the diagnosis, classification, and evaluation of treatment effectiveness. This need is particularly important in the case of ADHD, given the current questions and controversies regarding its nature, evaluation, and latent structure [30–32]. On the other hand, the DSM diagnostic criteria demands the presence of at least six symptoms within any of the two groups (inattention and hyperactivity/impulsivity) for an ADHD diagnosis in any of the subtypes of the disorder (presentations in the case of DSM-5). The addition of six symptoms to either of the two lists would lead to a diagnosis. In consequence, the DSM assumes that the 18 symptoms carry the same weight in an ADHD diagnosis and bear the same discriminatory capacity. However, it is reasonable to think that symptoms may differ in terms of severity and even in the reliability with they represent the disorder [18].

Therefore, to contribute to advance knowledge regarding the evaluation of this disorder, and to test the hypothesis that ADHD symptoms vary in severity and reliability, the current study seeks to validate and calibrate an ADHD symptomology scale using the graded response model (GRM, [33–34]), which is described in more detail below. Along with the partial credit model [35], GRM is one of the most often used systems for items with multiple categories of response, which (in principle) guarantees responses to all possible problems. Also, to our knowledge, no studies have used IRT analysis to assess symptoms of ADHD in a sample of Spanish children. Since cross-cultural replication of a model is fundamental requirement to assure its validity [36], one of our objectives is to contribute to fill this gap in research.

## Materials and Methods

### Participants

A total of 784 children participated (48.7% boys). These children were randomly selected at 5 schools (40 levels) in Spain. The mean ages of the boys and girls were 62 months (*SD* = 8.4 months) and 63 months (*SD* = 8.6 months), respectively. The participants were divided into age-based quartiles, and these groups did not differ by gender (X^2^_(3)_ = 3.54; *p* = 0.316). Regarding ethnicity, 95.6% of children were native country. Each teacher addressed about 20 children (M = 19.6) randomly recruited over the total of their level (M = 30.3). Because of confidentiality, we did not access to psychiatric diagnosis. All procedures performed in studies involving human participants were in accordance with the ethical standards of the institutional and/or national research committees and the 1964 Helsinki Declaration and its later amendments or comparable ethical standards. Also, written informed consent was obtained from all participants included in this study (teachers, and parents or legal guardians in the case of children), adjusted to the Code of Practice for Research at the University of Valladolid (Spain), and approved by the ethic committee of this University.

### Instrument

The “ADHD Questionnaire” [37] is a scale of 18 items composed by the symptoms proposed by the DSM-IV. The response options are divided into four frequencies (1 = *almost never*, 2 = *sometimes*, 3 = *often*, and 4 = *almost always*). The wording of items coincided with the Spanish version of the DSM-IV word for word, with two modifications: (a) the adverb “often” used at the beginning of the description of all symptoms was removed, and (b) all items were expressed in declarative tense to avoid response errors associated with formulating items in the negative. Two groups of items were considered for later analysis: The first ranged from 1 to 9 (symptomatology related to inattention; the IA subscale), and the second ranged from 10 to 18 (symptomatology related to hyperactivity/impulsivity; the HI subscale). The reliability and construct validity of the scale have been empirically supported by samples of Spanish children rated by parents and teachers [37–39].

### Procedure

Parents were informed in writing regarding the aims of the study, and their informed consent was obtained before their children were evaluated. A computerised version of the scale described above was created using LimeSurvey, v.1.9 (http://www.limesurvey.org/), and it was uploaded to an Internet server. We decided to use a computer-based evaluation which allows: (a) to standardize the procedure; (b) to avoid errors derived from manually coding the data; (c) to maximize confidentiality, and (d) to facilitate the teacher’s work. In addition, our decision was based on evidence showing that the test administration procedure does not affect the invariance of measurement (e.g., [40–43]).

The students’ teachers completed the scale over a 2-week period. After the time period ended, the data were gathered from the database, and the analysis was conducted. The raw data used in this research are provided as supporting material (S1 File).

### Data Analysis

The data were analysed using IRTPRO 1.0b [44], using the GRM [33–34]. Beyond the usual assumptions of IRT, the GRM assumes that the categories to which an individual responds (or to which he or she qualifies, as is the case here) can be ordered or placed on a hierarchy, for example, with probabilistic scales for summation estimates or Likert-type scales. The GRM attempts to gather more information than a scale with a dichotomous response (e.g., “yes” or “no”). In this sense, this model is an extension of the two-parameter logistic model (2-PLM) for multiple ordered categories and can be included among what are known as “difference models” [45]. These models are often considered as indirect IRT models, given that the calculation of conditional probability in which one responds to a determined category requires a two-step process.

The GRM specifies the probability of a person qualifying with a category *i*_k_ or above, as opposed to being included in a lower category when the rating system has at least three categories, is expressed as:

$$P_{ik}^{*}(\theta_{j})=\frac{e^{D_{\alpha i}(\theta_{j}-\beta_{ik})}}{1+e^{D_{\alpha i}(\theta_{j}-\beta_{ik})}}$$ (1)

$$P_{ik}(\theta_{j})=P_{ik}^{*}(\theta_{j})-P_{ik+1}^{*}(\theta_{j})$$ (2)

where *k* is the ordered response option; P_ik_(θ_j_) is the probability of responding with option *k* of item *i* with a latent trait level θ_j_; P*_ik_(θ_j_) is the probability of responding to option *k* or above of item *i* with a latent trait level θ_j_; θ_j_ is the latent trait level of the participant; β_ik_ is the localisation parameter of alternative *k* of item *i*; α_i_ is the discrimination parameter of item *i*; and *D* is the constant 1.702.

To ensure that the calibrated scale is useful, IRT requires that the empirical data fit the theoretical model. Although we do not have a universally accepted and unambiguous goodness-of-fit test [11, 30], we nevertheless offer some evidence, which according to the literature, suggests a good fit [46]. Thus, we examine how easy it is to reach convergence, the size of the standard errors, M_2_ and RMSEA statistics, the standardized local dependence statistics of each couplet of items, and the invariance of the discrimination and localisation parameters in two randomly selected subsamples. Local dependence was investigated using χ^2^ statistics from the observed versus expected frequencies in each of the two-way cross tabulations between responses to each item. Excessively large standardized χ^2^ values (i.e., >10) suggests a violation of the local independence assumption [46].

To estimate the fit of data to the GRM, M2 statistic and RMSEA (root mean square error of approximation) were computed. M2 is a Statistics based on one- and two-way marginal tables. Highly significant values from the M2 difference test indicate poor fit, especially when accompanied with high RMSEA values (>.08). On the other hand, a significative M2 statistic paired with a low RMSEA suggests the presence of a limited amount of model error, which is common in strong parametric models such as GRM [46].

We chose the GRM rather than other models such as the PCM [16] or successive intervals model [47] for the following reasons: First, the GRM was one of the first models developed to specifically analyse polytomous ordinal items; second, it is appropriate for items with different discrimination parameters; third, it is a natural model for cumulative sum estimation (i.e., Likert-type) scales; finally, numerous articles and other studies have been published regarding estimating parameters using the GRM model; thus, the conditions needed for obtaining correct estimates are well-known [48].

## Results

### Descriptive Statistics

The inattention (IA) subscale total score had a mean of 15.15 (SD = 5.89), and the hyperactivity-impulsivity (HI) subscale total had a mean of 16.46 (SD = 6.40). As expected [30], a sex comparison revealed significant differences: Boys scored higher on symptomology for both the IA (*F*_(1, 782)_ = 11.69, *p* = 0.001) and HI (*F*_(1, 782)_ = 5.122, *p* = 0.024) scales.

The item means ranged from 1.50 to 1.83 on the IA scale and from 1.35 to 2.11 on the HI scale, all of which were below the theoretical mean of the scale. We expected this result because the study used a non-clinical sample. The standard deviations ranged from 0.812 to 1.105 and were of equal magnitude for both scales. The mean of the corrected item-total correlations were *r* = 0.701 and *r* = 0.668 for the IA and HI scales, respectively.

### Uni-Dimensionality and Local Independence

Uni-dimensionality and local independence are basic requirements of IRT. Uni-dimensionality means that all items in a test measure one characteristic (i.e., a latent variable) of participants. Local independence means that the items are statistically independent beyond the latent variable being measured (in other words, the response to the item only depends on theta and other parameters, but not on the response to the other items. Importantly, “pure” uni-dimensionality is unfeasible. What IRT attempts, then, is to demonstrate the existence of a clearly dominant factor [35]. However, uni-dimensionality involves local independence but not the opposite [15]; thus, a demonstration of uni-dimensionality demonstrates local independence.

Three strategies were used to confirm uni-dimensionality: (a) a comparison of the proportion of variance explained by the first two factors; (b) the ratio between the individual values of factors 1 and 2; and (c) a parallel analysis. Table 1 shows the results of these analyses.

**Table 1:** Uni-dimensionality and Local Independence Indicators.

		IA	HI
PCA	Individual value F1 (%)	5.975 (59.4%)	5.714 (55.8%)
	Individual value F2 (%)	0.632 (7.9%)	0.859 (10.4%)
	Ratio F1/F2	9.454	6.652
	Mean item-total correlation	0.764	0.744
Correlations	IA teachers	1.000	
	HI teachers	0.687	1.000
Reliability	α Cronbach Ordinal (SE)	0.95 (0.22)	0.94 (0.24)
	θ ordinal (SE)	0.85 (0.39)	0.84 (0.40)

*Note*. IA = Inattention; HI = Hyperactivity/Impulsivity; PCA = Principal Component Analysis; SE = Standard Error of Measurement.

The proportions of the variance explained by the first factor for the IA and HI scales was 59.5% and 55.8%, respectively; thus, this factor surpasses the 20% proposed by Reckase [49] and the 40% proposed by Carmines and Zeller [50] by a wide margin. Furthermore, this result suggests the presence of a dominant factor responsible for item responses. This assertion is supported by high factor 1 loadings for all items (ranging from .72 to .86 on the IA scale and from .65 to .89 on the HI scale).

The ratios between the primary and secondary individual values were 9.45 and 6.65 for the IA and HI scales, respectively, surpassing the value of 5.0 suggested by Hambleton et al. [51], who dealt extensively with the uni-dimensionality of scales.

**Fig 1:**
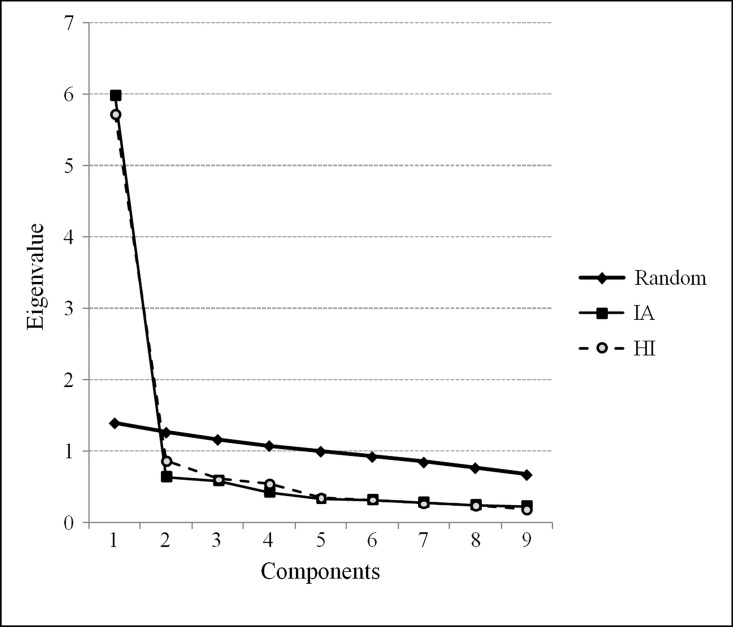
The results of a parallel analysis with 1,000 iterations of the IA and HI subscales.

A parallel analysis [52] was also conducted on the polychoric correlation matrix between the items to confirm the intersection of individual values obtained using the factorial solution with randomly obtained values. As Fig 1 shows, we can accept that an individual component for each of the two subscales is plausible, because the randomly generated individual values are greater than those generated by the analysed data after component 2.

The total-item mean correlations also support uni-dimensionality (*r* = .764 and *r* = .744 for the IA and HI scales, respectively). Lastly, the correlation between the subscale scores was significant (*r* = .687; *p* < .001). The results shown in the previous paragraphs provide sufficient evidence of the existence of a dominant factor in each scale, thereby enabling the use of IRT for data analysis.

### Parameter Estimation

**Fig 2:**
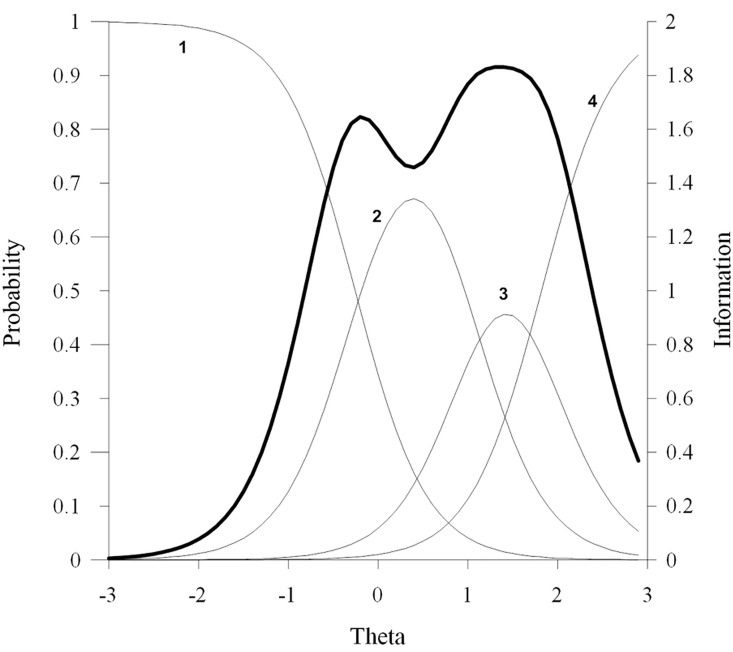
Characteristic response curves and the information curve for item 1 of the IA subscale.

To estimate the α_i_ and β_ik_ parameters for the items, the marginal maximum likelihood method was used. As an example, Fig 2 demonstrates the characteristic response curves for item 1 (“Fails to give close attention to details or makes careless mistakes in schoolwork or other activities”) on the IA scale. The horizontal axis represents the latent variable θ (*M* = 0; *SD* = 1). Four curves were drawn for each item, each of which represents the probability (represented on the vertical axis) of having one of the following response categories: *almost never* (1), *sometimes* (2), *often* (3), or *almost always* (4).

The thick lines in Fig 2 represent the item information function (IIF). The IIF is an index that shows the range of ability above θ for which the item is most useful when distinguishing among evaluated individuals. In other words, IIF characterises the precision of the measurement of children at different levels of the latent construct, such that the highest values indicate greater precision. For example, in the case of Fig 2, item 1 begins to offer the maximum amount of information for children with θ = 0.8, with information decreasing rapidly after θ = 1.8. It is within this latent variable (θ) range that the item becomes most useful.

Table 2 shows the values of parameters α_i_ and β_ik_. According to Baker [53], α_i_ values from 0.01 to 0.24 are very low; those from 0.25 to 0.64 are low; those from 0.65 to 1.34 are moderate; those from 1.35 to 1.69 are high; and those above 1.7 are very high. Therefore, we concluded that the discrimination patterns were either high or very high for all items on the two scales. The previous result was confirmed by the high correlation between parameters α_i_ and the corrected item-total correlations (*r* = .95 and *r* = .94 for the IA and HI scales, respectively).

**Table 2:** Estimations of the Discrimination (α_i_) and Localization (β_ik_) Parameters for ADHD Symptoms.

It	Content	Parameters			
		α (SE)	β_1_ (SE)	β_2_ (SE)	β_3_ (SE)
**Inattention subscale**					
IA1	Careless.	2.52 (.18)	-0.26 (.05)	1.04 (.04)	1.82 (.10)
IA2	Inattention.	2.05 (.16)	0.49 (.06)	1.55 (.10)	2.18 (.13)
IA3	Listen.	2.04 (.15)	-0.06 (.06)	1.36 (.09)	2.06 (.12)
IA4	Instruction.	2.63 (.19)	0.09 (.05)	1.33 (.08)	2.08 (.12)
IA5	Disorganized.	2.58 (.18)	0.02 (.05)	1.22 (.07)	2.03 (.11)
IA6	Avoid.	2.56 (.18)	0.05 (.05)	1.36 (.08)	1.92 (.11)
IA7	Lose.	1.77 (.14)	.049 (.06)	1.66 (.11)	2.42 (.16)
IA8	Distracted.	2.89 (.20)	-0.41 (.05)	0.89 (.06)	1.47 (.08)
IA9	Forgetful	2.55 (.18)	-0.01 (.05)	1.32 (.08)	1.91 (.11)
**Hyperactivity/Impulsivity subscale**					
HI1	Fidget.	2.53 (.18)	-0.31 (.05)	0.46 (.05)	1.16 (.07)
HI2	Seat.	3.11 (.22)	-0.38 (.05)	0.55 (.05)	1.23 (.07)
HI3	Run.	3.40 (.26)	0.03 (.05)	0.91 (.06)	1.53 (.08)
HI4	Quiet.	1.67 (.14)	1.03 (.08)	1.85 (.13)	2.66 (.19)
HI5	Motor.	2.57 (.19)	0.15 (.05)	0.91 (.06)	1.49 (.09)
HI6	Talk.	1.78 (.12)	-0.63 (.07)	0.71 (.07)	1.52 (.10)
HI7	Blurt.	1.45 (.11)	0.06 (.07)	1.59 (.11)	2.53 (.18)
HI8	Wait.	2.15 (.15)	-0.11 (.06)	1.06 (.07)	1.76 (.10)
HI9	Interrupt.	1.97 (.14)	-0.35 (.06)	1.08 (.07)	1.91 (.11)

*Note*. It = Item; IA = Inattention; HI = Hyperactivity/Impulsivity; SE = Standard Error of Measurement.

**Fig 3:**
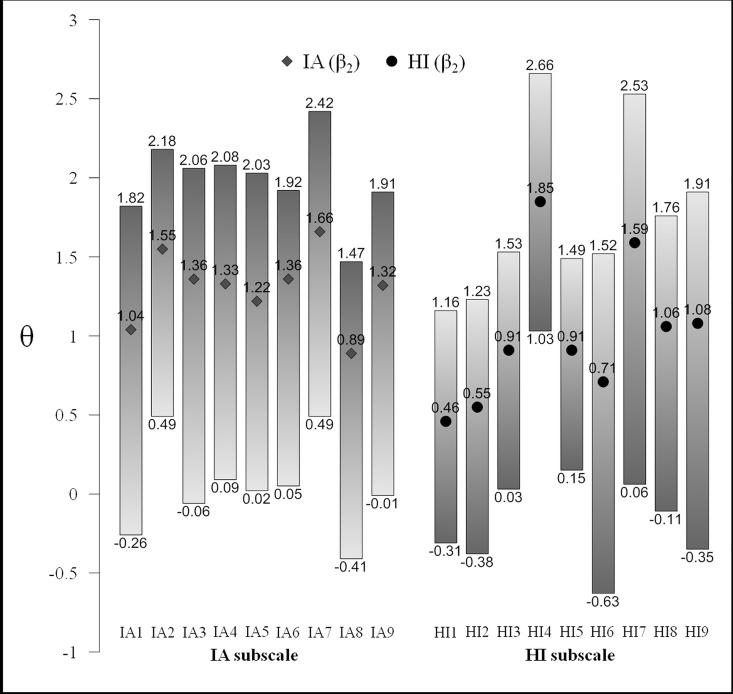
The magnitude of the β_ik_ localisation parameters.

The β_ik_ parameters ranged from approximately the mean to +2.5 *SD*s. The largest increments occurred between β_1_ and β_2_ (*M* = 1.16). The interval between β_2_ and β_3_ (*M* = 0.71) was lower. This transition presents less variability (SD = 0.11) than the first (SD = 0.24). Taken together, β_1_ values are situated approximately at the mean of the latent variable (*M* = -0.01); β_2_ values are situated at 1 *SD* above the mean (*M* = 1.16); and β_3_ values are situated at 2 *SD*s (*M* = 1.87) above the mean. Fig 3 provides a graphic representation of the localisation parameters for ease of interpretation.

For example, item HI4 “Difficulty playing or engaging in leisure activities quietly” shows the following values: β_1, 1_ = 1.03, β_1, 2_ = 1.85, and β_1, 3_ = 2.66. Thus, a relatively high trait level is needed to have a probability of .5 with regard to *almost never* responses, and a very high trait level is needed to reach a probability of .5 with regard to *almost always* responses. Conversely, item HI6 “Talks excessively” shows the following values: β_1, 1_ = -0.63, β_1, 2_ = 0.71, and β_1, 3_ = 1.52. Thus, an average trait level is needed to obtain a probability of .5 with regard to *almost never* responses, and a high trait level is needed for a probability of .5 with regard to *almost always* responses.

As shown in Fig 2, the slopes of the curves for each item were determined by the discrimination parameter (α_i_) such that step values (β_ik_) represented the intersection of each curve on scale θ. In the case of item 1, the most probable response for a θ score of -0.26 or below is *almost never*; if θ is between -0.26 and 1.04, then the most probable response is *sometimes*; if θ is between 1.04 and 1.82, then the most probable response is *often*; lastly, if θ is above 1.82, then the most probable response is *almost always* (these numerical values can be checked in Table 2). A correspondence can be observed between the highest values for the latent variable and the successive response categories. According to model expectations, one of the categories was the most probable at some point along the latent variable.

The correlation between the mean localisation parameter for each β_i, 2_ item and the item mean was *r* = -.97 for the IA scale and *r* = -.92 for the HI scale. Thus, greater item means denoted lower trait levels needed to qualify for one of the higher categories on the scale.

### Fitting the Model

First, convergence was reached on both scales in fewer than 30 iterations. Second, with regard to the α_i_ parameters on the IA scale, the standard error of the measurement (SE) range was *SE*_IA7_ = 0.14 to *SE*_IA8_ = 0.20; for the β_ik_ parameters, the SE range was *SE*_IA8,1_ = 0.05 to *SE*_IA7,3_ = 0.16. For the α_i_ parameters on the HI scale, the SE range was *SE*_HI7_ = 0.11 to *SE*_HI3_ = 0.26; for the β_ik_ parameters, the SE range was *SE*_HI1,1_ = 0.05 to *SE*_HI4,3_ = 0.19. Therefore, we conclude that the SE values were sufficiently reduced to suggest a good data fit. Third, the M_2_ statistics obtained for the IA scale (*M*_*2*_ = 707.94; *DF* = 315; p = .0001; *RMSEA* = .04) and the HI scale (*M*_*2*_ = 929.54; *DF* = 315; p = .0001; *RMSEA* = .05) indicate a lack of fit. Importantly, however, the associated *RMSEA* values of .04 and .05 might be because of the limited amount of “modelled error”, which leads us to conclude that the data fit might be sufficient if one considers the rest of the indices obtained. Third, we examined the standardized local dependence statistics for each couplet of items. In the case of IN, we found six pairs of items with a X2 value greater than 10 in five items which the observed covariation exceeded the covariation predicted by the model [54–55]: Listen/Inattention; Loses/Inattention; Distracted/Inattention; Loses/Instructions; Distracted/Instructions; Avoids/Loses; Forgetful/Loses. For HI the amount was five and two respectively (Fidgets/Blurts; Talks/Runs; Interrupts/Runs; Blurts/Quiet; Interrupts/Blurts). These results suggest certain local dependence between pairs of symptoms. Finally, we confirmed the invariance of the parameters with two randomly extracted subsamples. The sample was divided into two random subsamples of 397 participants, and the complete process for estimating α_i_ and β_ik_ parameters was repeated for both. Next, the correlations between the parameters were calculated. The correlations between the α_i_ discrimination parameters for the IA and HI scales were *r* = .86 (*p* < .01) and *r* = .92 (*p* < .01). The correlations between the β_ik_ parameters for the IA scale were β_i1_: *r* = .97 (*p* < .01); β_i2_: *r* = .87 (*p* < .01); and β_i3_: *r* = .96 (*p* < .01); the correlations between the β_ik_ parameters for the HI scale were β_i1_: *r* = .99 (*p* < .01); β_i2_: *r* = .97 (*p* < .01); and β_i3_: *r* = .94 (*p* < .01). These results provide evidence of the invariance of the parameters for both subscales.

### Measurement Precision

**Fig 4:**
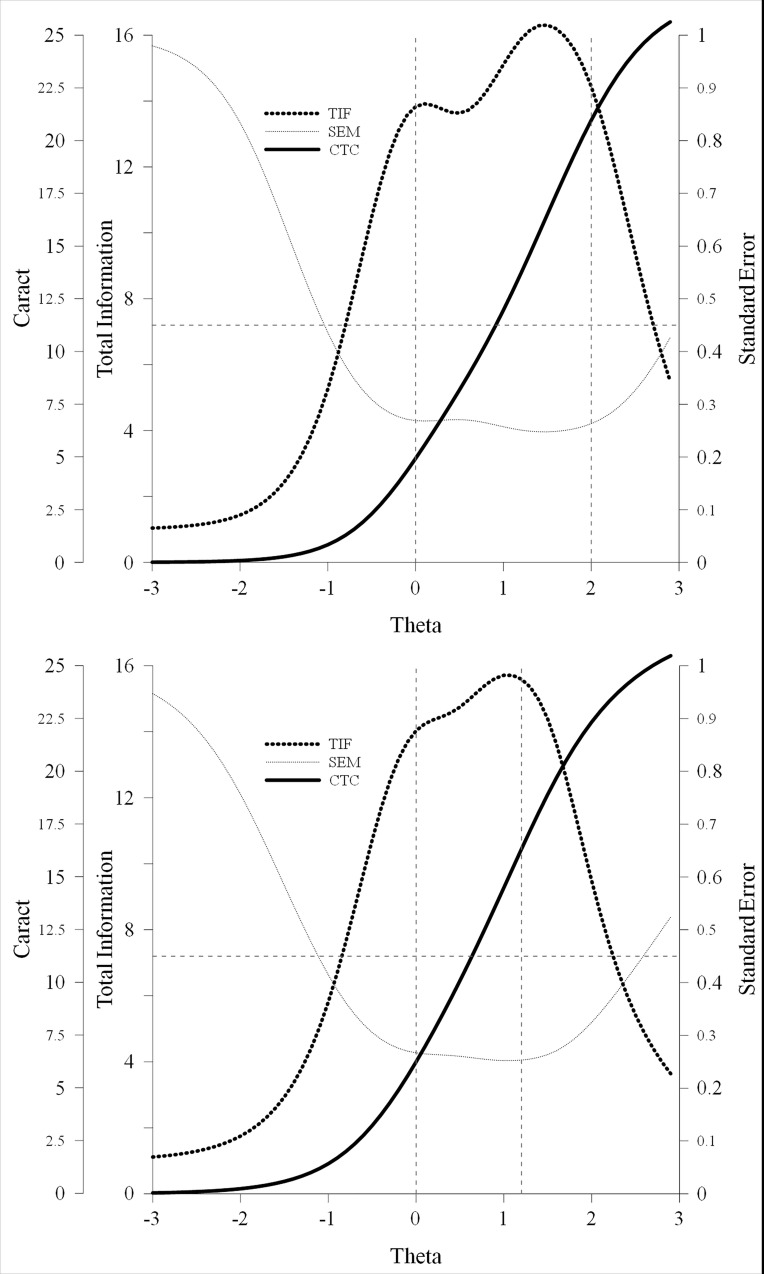
CTC, TIF, and the standard error of measurement (SE) of the IA and HI subscales.

The graphical representations of the characteristic test curve (CTC) and the test information function (TIF) are shown in Fig 4. The CTC for the IA scale shows a reasonable quantity of the latent trait from the mean and above, which is consistent with the β values (S1 Table). The thick dotted line represents the TIF (which is equivalent to the combined value of the information functions for the nine items). The test information values are greater for θ values between 0 and +2 SDs (i.e., 13.83 and 14.44). The lowest standard error values correspond to those with the greatest test information. As expected, the smaller SE values denote more information or precision in the scale with regard to latent θ. We found similar results for the CTC and the TIF for the HI scale. The CTC indicated a noticeable quantity of latent trait above the mean. The TIF indicates that the information values of the scale are greater for θ values between 0.0 and +1.2 SD (i.e., 14.03 and 13.61). In summary, both subscales reach their maximal accuracy between the mean and a relatively high region of the latent variable. The discriminative capability of scores decreases very fast in the high regions of the trait, and also immediately below the mean. This result suggest that, at least in this kind of sample, the scale is adequate for screening purposes (i.e., to identify subjects with higher trait levels), but it is probably less useful to identify differences between children with high ADHD levels.

Vertical dotted lines represent the area of θ where the scale measures have greater precision.

In summary, the results described above indicate the following:

1. All of the items in both subscales showed movement toward higher levels of the latent variable as the response categories increased.
2. The discrimination patterns were elevated in all cases, which indicate that the items behave properly when being discriminated between different levels of the latent variables “inattention” and “hyperactivity/impulsivity.” This finding also indicates that the items were defined with sufficient clarity.
3. The best symptoms for discriminating the levels of these traits by teachers included in the IA subscale were items IA8 (“Easily distracted by extraneous stimuli”) and IA4 (“Does not follow through on instructions and fails to finish schoolwork, chores, or duties in the workplace”). The items with more discriminatory power for the HI subscale were items HI2 (“Leaves seat in classroom or in other situations in which remaining seated is expected”) and HI3 (“Runs about or climbs excessively in situations in which it is inappropriate”).
4. Despite the degree of observed variability between the symptoms in both subscales, nearly all of the cases confirmed an extremely low representation of the latent variable in the levels below the mean, which suggests a questionable degree of reliability for symptoms with latent scores below this point. We believe this circumstance was to be expected, given the non-clinical nature of the sample. This finding suggests that future studies using samples with these characteristics should involve participants from clinical populations. Likewise, a reasonable quantity of the trait between the median and +1.6 SDs was observed. In all cases, the most likely responses in these areas were 3 and 4 (and not 1 and 2).
5. The IIF symptom score approximating +2.8 SDs was low; thus, latent scores above that point have low reliability. The greatest reliability value was observed for trait levels falling between the mean and +1.5 SDs.

## Discussion

This study analysed the psychometric properties of two evaluation scales for ADHD symptomology based on DSM-IV-TR criteria as assessed by teachers. Based on the results obtained, we determine that the IA and HI scales comprise a significant portion of the measurable aspects of the syndrome according to the standards established by the DSM-IV-TR. Because a universally accepted fit index has not been created for the GRM, a set of evidence can be used to determine whether the fit is acceptable. Our set included the ease of reaching convergence, reaching reasonable and interpretable estimated parameters based on the theory used to build the instrument, low standard error magnitudes for the estimated parameters, parameter invariance, and confirming to diagnostic statistics. All of the above criteria were sufficiently met; thus, the data showed a sufficiently good fit to the GRM.

Using different procedures, we provided evidence of meeting the uni-dimensionality and local independence requirements. In each of the analysed scales, a dominant factor justifies IRT analysis. The current study might also have implications regarding the use of the classification scales evaluating ADHD symptomology, given that it has solid psychometric properties that allow us to make the following conclusions:

First, the DSM-IV-TR demands the presence of at least six symptoms within any of the two groups for an ADHD diagnosis. The addition of six symptoms to either of the two lists would lead to a diagnosis of one of the subtypes/presentations of ADHD. Added to this decision criterion is the acceptance that the 18 symptoms carry the same weight in an ADHD diagnosis and bear the same discriminatory capacity. However, relative differences exist in both the position of the symptoms relative to the measured variable (a position that can be interpreted as relative symptom severity, such that more difficult items are signs of a more severe syndrome) and in differences in informativeness and reliability. This finding suggests that it would be useful to identify the symptoms that are more important than the others with regard to diagnosing ADHD and its subtypes [25].

Second, although all items have demonstrated psychometric properties within the acceptable range, certain items have lower discrimination values than the others, as well as poorer informational qualities and lower reliability values at all levels with regard to the respective latent traits. In the case of the IA scale item “Loses things necessary for task or activities”, prior evidence shows its lower informative capacity [5]. Regarding the possible implications for the review of ADHD symptomology, the symptoms “Loses things necessary for task or activities” on the IA scale and “Talks excessively” and “Blurts out answers before questions have been completed” on the HI scale present relatively low discrimination values than the other symptoms as well as poor information and the lowest reliability values of all of the levels for the respective latent traits. These results partially replicate previous findings revealing relatively low discriminative values for the symptoms “loses things” [5, 8, 22, 26, 29], “talks excessively” [5, 8, 22, 24] and “blurts answers” [8, 24, 29]. Nevertheless, relatively high levels were maintained. As will be discussed next, this finding suggests the need for changes in how these items are written to improve their informational capacity and reliability. In the DSM-5, the symptoms of ADHD basically kept the same wording as in DSM-IV, except the examples added in some of them. An interesting target for future research is to determine whether those examples effectively improve the symptoms (items) discrimination.

These findings also support the appropriateness of reducing the list of symptoms to just include those with greater discriminatory capacity, thereby resulting in a more parsimonious diagnostic process. Additionally, based on recent evidence showing that some symptoms do have more predictive power on both diagnosis and impairment level [29], our results suggest that reducing the scale without losing relevant information would allow it usage as a screening tool. Interestingly, previous studies reveal that certain symptoms (e.g. instructions) tend to have adequate discrimination levels regardless the kind of assessed samples (general or clinical samples). At the same time, as we observed, other items, like “blurt” and “talks”, tend to have low discriminative parameters and local dependence, which supports evidence recently published [8]. In our view, this knowledge could be a starting point to create brief but highly sensitive screening measures for ADHD.

Third, in almost all of the cases, a very low representation of the latent trait was found for levels below the mean as well as for very high levels of the latent variable. This finding does not invalidate the diagnostic capacity of the DSM symptoms. This result is similar to previous studies addressing general samples [5, 22, 24]. Nevertheless, the low reliability of the scores for the low and high-average variable measures might have implications for studies dedicated to a) analysing the ADHD structure in the general population using a dimensional symptom perspective; b) analysing ADHD symptomology and their subtypes in a clinical population where one presumes that high and very high levels of the latent variable would be observed; and c) precisely discriminating the degree of severity among children who have already been diagnosed; the latter is specially relevant in the context of previous evidence revealing that some items can be diagnostically sensitive across general and clinical samples [8]. Thus, our results suggest the need to elaborate a set of appropriate indicators for very high levels of the latent variable, especially when the aim is to precisely estimate the trait in participants with an elevated degree of ADHD.

Finally, it is necessary to keep certain study limitations in mind to sufficiently interpret the current results and indicate possible lines of future studies. First, the psychometric properties of the items were calculated based on teacher observations; it would have been better to compare them with parental scores because similar studies have observed differences in the usefulness of certain symptoms depending on the informant [5]. Second, it would have been better to use clinical samples to determine the function of the items and the test itself based on elevated levels of severity and in relation to specific ADHD subtypes. Third, our results relate to a scale directly derived from the 18 symptoms listed in the DSM. These results need to be replicated in other rating scales with different number of symptoms or with different wording. Fourth, the information about the diagnosis of both psychiatric symptoms and other conditions (e.g., learning difficulties) was not available. To include this information in future research would be useful to establish the cut-off point of the continuous latent where behaviours can be considered pathologic. Fifth, although it is not a limitation of the present study, given the new differential diagnostic criteria of the DSM-5, it is necessary to test the functioning of the scale in specific populations (e.g., autistic spectrum disorders).

Our data suggest, in line with previous studies, that ADHD symptoms vary in severity and in the amount of information they provide for evaluation of the disorder. These results question the utility of the DSM symptom criteria, where it is assumed that any combination of six symptoms in either of the two categories is valid for diagnosis. Thus, our results suggests the possibility of detecting symptoms that are more relevant than others for diagnosis. According to Li et al. [8], an interesting line of research is the development of new diagnostic algorithms, weighting each symptom according to their ability to discriminate individuals with high levels in the ADHD continuum. (ie, potential diagnoses), in order to achieve more precise assessments. However, achieving this objective requires further investigation with large community and clinical samples. In addition, given the developmental heterogeneity of ADHD [32], symptoms may vary in the amount of information they provid to the diagnosis along different ages. Thus, longitudinal IRT studies are needed. Using an IRT framework for the study of ADHD can provide valuable information for the assessment and diagnosis of the disorder. However, IRT studies are still relatively scarce, so researchers are encouraged to use these models.

## Supporting Information

S1 Fig Characteristic response curves and the information curve for 9 items of the IA subscale. (TIFF)

S2 Fig Characteristic response curves and the information curve for 9 items of the HI subscale. (TIFF)

S1 File Excel file with the raw data used in this research. (RAR)

S1 Table Information function of items for ADHD symptoms at different levels of the latent trait. (DOCX)
