# Supplementary figures and images for: Hierarchy and Psychometric Properties of ADHD Symptoms in Spanish Children: An Application of the Graded Response Model
Source: PLoS One. 2016 Oct 13;11(10):e0164474. doi: 10.1371/journal.pone.0164474 (PMC5063325; doi:10.1371/journal.pone.0164474)

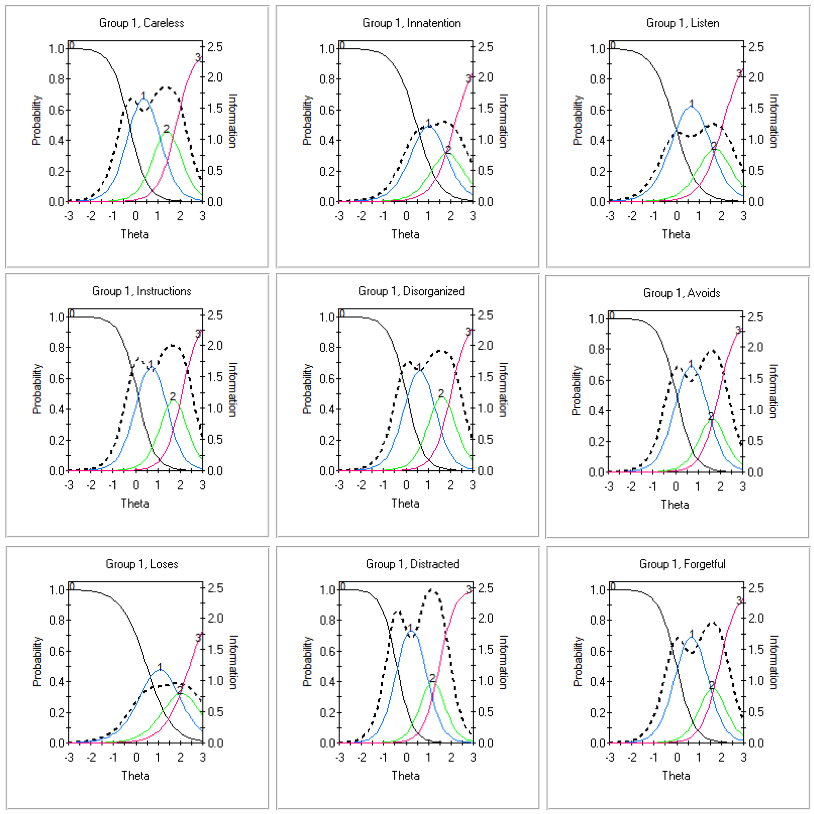

Supplement: S1 Fig — (TIFF) [file pone.0164474.s001.tiff]

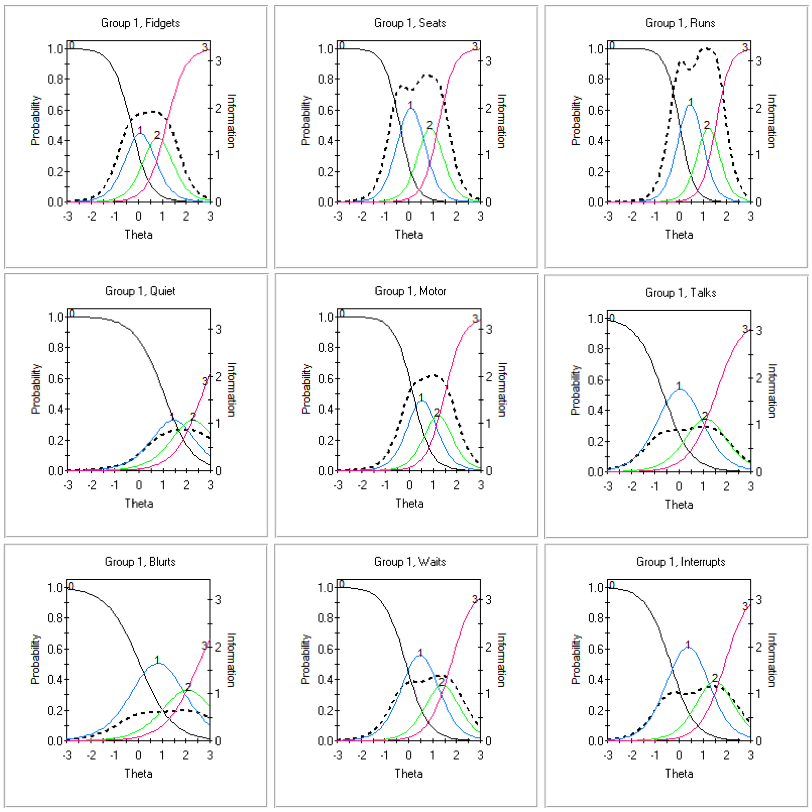

Supplement: S2 Fig — (TIFF) [file pone.0164474.s002.tiff]
