# Supplementary material for: Hierarchy and Psychometric Properties of ADHD Symptoms in Spanish Children: An Application of the Graded Response Model
Source: PLoS One. 2016 Oct 13;11(10):e0164474. doi: 10.1371/journal.pone.0164474 (PMC5063325; doi:10.1371/journal.pone.0164474)
Supplement: S1 Table — (DOCX) [file pone.0164474.s004.docx]

S1 Table. Information Function of Items for ADHD Symptoms at Different Levels of the Latent Trait.

| It | Content | Estimated θ | | | | | | | | | | | | | | |
| --- | --- | --- | --- | --- | --- | --- | --- | --- | --- | --- | --- | --- | --- | --- | --- | --- |
|  |  | -2.8 | -2.4 | -2.0 | -1.6 | -1.2 | -0.8 | -0.4 | 0.0 | 0.4 | 0.8 | 1.2 | 1.6 | 2.0 | 2.4 | 2.8 |
| **Inattention subscale** | |  |  |  |  |  |  |  |  |  |  |  |  |  |  |  |
| IA1 | Careless. | 0.01 | 0.03 | 0.08 | 0.20 | 0.49 | 1.03 | 1.56 | 1.60 | 1.46 | 1.65 | 1.82 | 1.81 | 1.57 | 0.97 | 0.46 |
| IA2 | Inattention. | 0.00 | 0.01 | 0.03 | 0.03 | 0.12 | 0.26 | 0.50 | 0.84 | 1.11 | 1.19 | 1.22 | 1.27 | 1.25 | 1.06 | 0.73 |
| IA3 | Listen. | 0.02 | 0.03 | 0.08 | 0.17 | 0.34 | 0.62 | 0.94 | 1.10 | 1.06 | 1.06 | 1.17 | 1.25 | 1.19 | 0.96 | 0.63 |
| IA4 | Instruction. | 0.00 | 0.01 | 0.03 | 0.08 | 0.22 | 0.56 | 1.18 | 1.74 | 1.70 | 1.60 | 1.88 | 2.00 | 1.94 | 1.48 | 0.79 |
| IA5 | Disorganized. | 0.00 | 0.01 | 0.04 | 0.10 | 0.26 | 0.64 | 1.26 | 1.72 | 1.65 | 1.64 | 1.87 | 1.90 | 1.83 | 1.35 | 0.71 |
| IA6 | Avoid. | 0.00 | 0.01 | 0.03 | 0.09 | 0.25 | 0.61 | 1.21 | 1.67 | 1.57 | 1.48 | 1.77 | 1.95 | 1.76 | 1.17 | 0.57 |
| IA7 | Lose. | 0.01 | 0.02 | 0.04 | 0.07 | 0.14 | 0.26 | 0.45 | 0.66 | 0.83 | 0.89 | 0.91 | 0.94 | 0.95 | 0.89 | 0.73 |
| IA8 | Distracted. | 0.01 | 0.03 | 0.08 | 0.25 | 0.70 | 1.54 | 2.13 | 1.82 | 1.75 | 2.27 | 2.46 | 2.13 | 1.24 | 0.50 | 0.17 |
| IA9 | Forgetful | 0.01 | 0.01 | 0.04 | 0.11 | 0.28 | 0.67 | 1.28 | 1.68 | 1.53 | 1.48 | 1.78 | 1.94 | 1.73 | 1.14 | 0.55 |
| Test information: | | 1.07 | 1.17 | 1.44 | 2.13 | 3.81 | 7.18 | 11.51 | 13.83 | 13.66 | 14.27 | 15.88 | 16.20 | 14.44 | 10.53 | 6.33 |
| Expected SE: | | 0.97 | 0.92 | 0.83 | 0.69 | 0.51 | 0.37 | 0.29 | 0.27 | 0.27 | 0.26 | 0.25 | 0.25 | 0.26 | 0.31 | 0.40 |
| Marginal reliability: | | 0.86 |  |  |  |  |  |  |  |  |  |  |  |  |  |  |
| **Hyperactivity/Impulsivity subscale** | |  |  |  |  |  |  |  |  |  |  |  |  |  |  |  |
| HI1 | Fidget. | 0.01 | 0.03 | 0.09 | 0.22 | 0.55 | 1.12 | 1.68 | 1.87 | 1.92 | 1.90 | 1.74 | 1.22 | 0.62 | 0.26 | 0.10 |
| HI2 | Seat. | 0.01 | 0.02 | 0.06 | 0.21 | 0.64 | 1.61 | 2.51 | 2.44 | 2.63 | 2.77 | 2.65 | 1.78 | 0.74 | 0.24 | 0.07 |
| HI3 | Run. | 0.00 | 0.00 | 0.01 | 0.05 | 0.17 | 0.62 | 1.78 | 2.99 | 2.84 | 3.16 | 3.30 | 3.01 | 1.63 | 0.54 | 0.15 |
| HI4 | Quiet. | 0.00 | 0.01 | 0.02 | 0.03 | 0.06 | 0.12 | 0.21 | 0.36 | 0.54 | 0.72 | 0.82 | 0.86 | 0.86 | 0.83 | 0.75 |
| HI5 | Motor. | 0.00 | 0.01 | 0.03 | 0.07 | 0.19 | 0.49 | 1.05 | 1.67 | 1.91 | 1.98 | 1.99 | 1.74 | 1.13 | 0.54 | 0.22 |
| HI6 | Talk. | 0.06 | 0.13 | 0.24 | 0.41 | 0.63 | 0.81 | 0.87 | 0.87 | 0.91 | 0.95 | 0.95 | 0.87 | 0.68 | 0.46 | 0.27 |
| HI7 | Blurt. | 0.03 | 0.06 | 0.10 | 0.16 | 0.25 | 0.37 | 0.48 | 0.56 | 0.59 | 0.60 | 0.62 | 0.63 | 0.64 | 0.61 | 0.54 |
| HI8 | Wait. | 0.01 | 0.03 | 0.08 | 0.17 | 0.37 | 0.70 | 1.07 | 1.26 | 1.25 | 1.31 | 1.39 | 1.36 | 1.14 | 0.75 | 0.41 |
| HI9 | Interrupt. | 0.03 | 0.07 | 0.14 | 0.28 | 0.52 | 0.81 | 1.01 | 1.02 | 0.99 | 1.06 | 1.14 | 1.15 | 1.04 | 0.79 | 0.49 |
| Test information: | | 1.17 | 1.35 | 1.75 | 2.60 | 4.38 | 7.64 | 11.67 | 14.03 | 14.58 | 15.44 | 15.59 | 13.61 | 9.47 | 6.03 | 3.99 |
| Expected SE: | | 0.93 | 0.86 | 0.76 | 0.62 | 0.48 | 0.36 | 0.29 | 0.27 | 0.26 | 0.25 | 0.25 | 0.27 | 0.32 | 0.41 | 0.50 |
| Marginal reliability: | | 0.87 |  |  |  |  |  |  |  |  |  |  |  |  |  |  |
